# Supplementary material for: Bat species assemblage predicts coronavirus prevalence
Source: Nat Commun. 2024 Apr 4;15:2887. doi: 10.1038/s41467-024-46979-1 (PMC10994947; doi:10.1038/s41467-024-46979-1)
Supplement: Supplementary file 1 — Supplementary Information [file 41467_2024_46979_MOESM1_ESM.pdf]

Supplementary Material for “Bat species assemblage predicts coronavirus prevalence”

Magdalena Meyer; Dominik W. Melville; Heather J. Baldwin; Kerstin Wilhelm; Evans Ewald Nkrumah; Ebenezer K. Badu; Samuel Kingsley Oppong; Nina Schwensow; Adam Stow; Peter Vallo; Victor M. Corman; Marco Tschapka; Christian Drosten; Simone Sommer

1. Supplementary Material and Methods

Details on fieldwork and sampling protocol

Cave features and anthropogenic disturbance

Details on sample selection and cytochrome b sequencing

Details on RNA purification and CoV characterization

Phylogenetic analysis of CoVs

2. Supplementary Results

3. Supplementary Reference List

1. Supplementary Material and Methods

Details on fieldwork and sampling protocol

As part of a large-scale longitudinal study to uncover bat-associated viral diversity in Ghanaian chiropterans, 14,464 bats were captured across 17 sampling sites in Ghana, West Africa between August 2010 to August 2012. Sampling sites were day roosts located in caves, abandoned mines or buildings in semi-forested farming land in close proximity to human settlements. Bats were also caught opportunistically in open forests or farmland (see Supplementary Table 1 for details). While most sites were sampled twice per year or even more sporadically, five cave sites in central Ghana represented core sampling sites (Buoyem 1 (N7°72'35.833" W1°98'79.167), Buoyem 2 (N7°72'38.056" W1°99'26.389), Forikrom (N7°58'97.5" W1°87'30.299), Kwamang 1 (N6°58'0.001" W1°16'0.001) and Kwamang 2 (N7°43'24.899" W1°59'16.501) (Fig. 1)). These core cave sites were sampled every two months (Supplementary Table 1). Caves were sampled using nylon-mist nets (6, 10 and 12m in length) strung along the cave entrances one hour after dusk until dawn. The number of mist nets depended on the width and shape of the cave entrances, ranging between 1 and 3 nets per cave. Further details on capture protocols can be found elsewhere[1].

Supplementary Table 1. List of 17 sampling sites during the two-year sampling effort in Ghana, West Africa. Bats were captured in caves, abandoned mines or buildings, and in open areas on forested or farmed land. While core sites were sampled every two months, other sites were only sampled twice a year or opportunistically.

Site	Site type	Latitude	Longitude	Sampling regime	# of captured individuals
**Akpafu Todzi**	mine	7.2619722	0.4915278	semi-annual	1470
**Bobiri**	open	6.6871	-1.34415	opportunistic	50
**Botanical Gardens**	open	6.6851111	-1.5618889	opportunistic	20
**Lake Bosomtwe**	open	6.5395278	-1.4115278	opportunistic	3
**Buoyem 1**	cave	7.7235833	-1.9879167	bimonthly	1667
**Buoyem 2**	cave	7.7238056	-1.9926389	bimonthly	2529
**Elmina**	building	5.0827778	-1.3483056	semi-annual	927
**Forikrom**	cave	7.58975	-1.8750833	bimonthly	1928
**KCCR***	open	6.6698226	-1.5771767	opportunistic	22
**Kwamang 1**	cave	7.0035685	-1.3003098	bimonthly	2811
**Kwamang 2**	cave	6.9832778	-1.2731944	bimonthly	1975
**Kwamang 3**	open	7.0065315	-1.3012354	opportunistic	20
**Likpe Todome 1**	cave	7.1639444	0.6079167	semi-annual	513
**Likpe Todome 2**	Cave	7.1638611	0.6081389	semi-annual	153
**Shai Hills**	Cave	5.9290000	0.0750000	opportunistic	41
**University Cape Coast**	Open	5.1202112	-1.2935219	opportunistic	321
**Bui**	Open	8.3893458	-2.3813621	opportunistic	15

*Kumasi Centre for Collaborative Research in Tropical Medicine

Captured bats were measured for weight and forearm length, sex, age and species identity based on morphological criteria was assigned if possible. In order to allow for the potential of recapturing, bats were tagged with a metal ring bearing a unique identifier. Over the course of the entire sampling period 2.4% of the bats were recaptured. The majority of cave dwelling bats belonged to the cryptic *Hipposideros caffer* complex, which comprises at least three lineages (*Hipposideros caffer B, C* or *D*) in this region[2]. Sequencing of the *cytochrome b* (*cytb*) gene of a small subset (n=267) of individuals had previously confirmed the presence of all three lineages at our study sites[3]. Owing to the presence of these cryptic species a minimally invasive wing punch (2mm) was taken and stored in -20 °C to allow for *cytb* typing. Lastly, faecal samples were successfully collected from 13,051 bats (out of the 14,464 bats in Supplementary Table 1) and used to screen for possible coronavirus (CoV) infections. The sampling was conducted consistently across sites and sampling events. Each bat was held in

individual bags, and faecal samples were retrieved as quickly as possible, within a maximum time of up to 2 hours[1]. The samples were then preserved in RNAlater at room temperature and promptly transferred to -80 °C for long-term storage (Qiagen, Germany). The frozen samples remained at -80 °C throughout the transfer process, ensuring an uninterrupted cold chain.

Cave features and anthropogenic disturbance

The geophysical features of the studied caves and the associated anthropogenic threats were evaluated using the Biotic Vulnerability (BV) scale for our study sites by Nkrumah et al., 2021 based on the publication of Tanalgo et al., 2018[1,4] (Supplementary Table 2). This scale considers factors such as cave morphology, accessibility, tourism potential, guano exploitation, and the presence of temples or shrines. To determine the BV of each cave, the total scores obtained from these features and activities are divided by the total number of factors assessed. This calculation yields average scores for each cave site based on predefined parameters, which are then interpreted within a specified range of mean scores. The BV index value is subsequently translated into a corresponding status, describing the importance and risk level associated with the cave's biota. It ranges from a minimum value of 1.00, corresponding to 'Status A' (indicating a highly disturbed and/or prone-to-disturbance cave), to a maximum value of 4.00, representing 'Status D' (indicating pristine caves with no disturbance).

1–1.99 - A Greater accessibility and highly prone to human disturbance and activities.
2–2.99 - B Lesser accessibility but disturbance is/may be present in distance.
3–3.99 - C Less accessibility, less prone to human disturbance.
4.00 - D No disturbance, far from localities and difficult to pass.

In conjunction with the Biotic Potential Index (BP), which encompasses factors such as species richness, abundance, relative abundance, and species attributes like endemism and conservation status (sourced from the World Conservation Union (IUCN) at www.iucnredlist.org), it is possible to calculate the Bat Cave Vulnerability Index (BCVI). This method is tailored for prioritizing tropical bat caves and assessing the conservation needs of

the caves under study. The BCVI combines the BP and BV components to establish a cave prioritization system: caves rated as 1A, 1B, and 2A are considered of the highest priority; those rated as 1C, 1D, 2B to 3D fall into the medium priority category; and caves classified as 4A-4D indicate a lower requirement for conservation measures. For further information on criteria, a comprehensive understanding of conservation priorities, and detailed guidance on BCVI utilization, please see Tanalgo et al., 2018[4] and Tanalgo et al., 2022[5].

Supplementary Table 2. Conservation priority level of the five studied caves adapted from Nkrumah et al., 2021[1]. Categorisation was based on Tanalgo et al., 2018[4].

Cave	Biotic Vulnerability Scores	Biotic Vulnerability Index	Bat Cave Vulnerability Index	Priorities
**Buoyem 1**	1.6	B	3B	Medium
**Buoyem 2**	1.6	B	2B	Medium
**Forikrom**	1.3	A	3A	Medium
**Kwamang 1**	1.3	A	2A	High
**Kwamang 2**	1.7	B	3B	Medium

The interaction between humans and bats within and around the studied bat roosting caves was examined by Anti et al., 2015[6]. The data was gathered through focus group discussions and stratified household surveys conducted in the communities of Buoyem, Forikrom, and Kwamang in Ghana from 2011 to 2012 (Supplementary Table 3).

Supplementary Table 3. Purposes of cave visitations in the communities of Buoyem, Forikrom and Kwamang, Ghana, adapted from Anti et al., 2015[6].

	Buoyem n = 412	Forikrom n = 362	Kwamang n = 500
**Respondents visiting bat caves**	181 (43.9)	178 (49.3)	222 (44.4)
**For religious activities**	19 (4.6)	79 (21.8)	5 (1)
**For recreation**	58 (14.1)	73 (20.2)	46 (9.2)
**To collect bat guano**	0 (0)	14 (3.9)	2 (0.4)
**To fetch water**	1 (0.2)	0 (0)	123 (24.6)
**To hunt for bats**	102 (24.8)	6 (1.7)	10 (2)
**To farm**	9 (2.2)	17 (4.7)	33 (6.6)
**For other reasons**	2(0.5)	5 (1.4)	5 (1.4)

The anthropogenic impact on the surrounding landscape of the studied cave locations, examining the intensity of stressors such as agriculture, urbanization, human intrusion, and transportation was performed by Theobald et al. 2020[7,8], and provides an assessment of human-induced modifications at regional scales in 2010 (Supplementary Table 4).

Supplementary Table 4. Overview of anthropogenic stressors for the surrounding area of the studied cave locations in 2010 at a 300 m resolution adapted from Theobald et al., 2020[7,8]. The degree of human modification (HM) is calculcated based on several stressor groups including AG agriculture/timber harvest (AG), urban and built-up areas (BU), human intrusion (HI), and transportation and infrastructure (TI).

Location	HM	AG	BU	HI	TI
**Bouyem**	**0.2418**	0.1833	0.0124	0.043	0.002
**Forikrom**	**0.2413**	0.1325	0.0089	0.055	0.018
**Kwamang**	**0.3849**	0.2847	0.0095	0.088	0.000

Details on sample selection and cytochrome b sequencing

Based on the capture information, irrespective of whether species identity was resolved or not, we selected a proportionate number of samples from each location and time point. This stratified subsampling approach contained 2,362 bats (of which 0.25% were recaptures). This subset contained 1,172 bats of the *Hipposideros caffer* complex and required us to genotype the *cytb* region – a highly conserved site amongst mammals[9] – to assign each individual to their respective *Hipposideros caffer* lineage.

DNA was first extracted from wing punches using an ammonium acetate protocol[10]: 250 µL Digsol buffer, 10 µL proteinase K (10mg/ml), and 10 µL dithiothreitol, or DTT (0.1M) were added to each sample. The tubes were kept overnight in a thermoshaker at 56°C and 800 rpm. Then 300 µL 4M ammonium acetate solution was added and the tubes left on a thermoshaker at 25°C and 800 rpm for 30 min to precipitate proteins. Next, the samples were centrifuged at 13,000 rpm for 10 min at room temperature, the supernatant transferred to new 1.5 mL tubes, and 550 µL 100% isopropanol (stored at -20°C) and 1.25 µL glycogen solution (20 mg/mL)

were added, before manually mixing the sample by gentle inversion. The tubes were then stored overnight at -20°C. Following centrifugation at 13,000 rpm for 30 min at 4°C, the liquid was carefully decanted and 500 µL 80% ethanol were added to each tube, before gently inverting the tube to mix the liquids. The tubes were centrifuged at 13,000 rpm for 30 min at 4°C. The alcohol was carefully decanted and the tubes were placed inverted and with the lids open to dry. Another 40 µL TE buffer was added to each dry tube. Subsequently, the tubes were left on a thermoshaker at 37°C and 500 rpm for one hour to dissolve the pellet, and stored at -20°C. The *cytb* locus of the *Hipposideros caffer* samples was amplified using the following PCR protocol. PCR was performed in a 10 µL reaction mixture, which contained 0.3 µM of one of the forward primers, 0.3 µM of the reverse primer (Supplementary Table 5), 1 µL DNA, 3.4 µL ddH_2O, and 5.0 µL AmpliTaq Gold^{TM} 360 Master Mix (Applied Biosystems, Germany). Thermocycling started with an initial denaturation step at 95°C for 10 min. This was followed by 30 cycles of denaturation at 95°C for 30 s, annealing at 57°C for 30 s, and elongation at 72°C for 30 s, and a final extension at 72°C for 2 min. The PCR products along with a ladder were run on a 1.5% TBE agarose gel via gel electrophoresis at 110 V for 30 min and visualized to ensure PCR had successfully amplified fragments matching the expected size (of 190 bp) of the *cytb* region.

Supplementary Table 5. Primers used to amplify the *cytb* locus in *Hipposideros* bats.

**Primer**	**Direction**	**5' to 3' Sequence**
CS1_NNNN_BC1_L15255	Forward	ACACTGACGACATGGTTCTACANNNNACAACTGCAAGTAGACAAAGCCACCCTHAC
CS1_NNNN_BC2_L15255	Forward	ACACTGACGACATGGTTCTACANNNNATTAGCGAGTGTAGACAAAGCCACCCTHAC
CS1_NNNN_BC3_L15255	Forward	ACACTGACGACATGGTTCTACANNNNACAACGAACAGTAGACAAAGCCACCCTHAC
CS1_NNNN_BC4_L15255	Forward	ACACTGACGACATGGTTCTACANNNNAGAGCGCCAAGTAGACAAAGCCACCCTHAC
CS1_NNNN_BC5_L15255	Forward	ACACTGACGACATGGTTCTACANNNNAGGTAGCTCAGTAGACAAAGCCACCCTHAC
CS1_NNNN_BC6_L15255	Forward	ACACTGACGACATGGTTCTACANNNNAACGCCAAGAGTAGACAAAGCCACCCTHAC
CS2-H15344	Reverse	TACGGTAGCAGAGACTTGGTCTYGATGGRATTCCTGTTGGR

Aliquots of the PCR products were further barcoded using a protocol adapted for the Illumina sequencing platform: first, the six forward and single reverse primers were tagged to Fluidigm adapters CS1 and CS2 (Access Array System™ for Illumina Sequencing System, ©Fluidigm, USA). Four additional, random bases after the CS1 adapter supports cluster identification during the Illumina run. Furthermore, a 10bp barcode (BC1-6) was attached to the forward primer. PCR was performed in a 20 µL reaction mixture, which contained 2.0 µL DNA, 4.0 µL barcode primers (Access Array Barcode for Illumina Sequencer, Single Direction, Fluidigm USA), 4.0 µL ddH2O, and 10.0 µL AmpliTaq Gold™ 360 Master Mix. Thermocycling began with an initial denaturation step at 95°C for 10 min, followed by 10 cycles of denaturation at 95°C for 30 s, annealing at 60°C for 30 s, and elongation at 72°C for 30 s, and a final extension at 72°C for 2 min. Thereafter, the barcoded samples were cleaned on a GeneTheatre robot (Analytik Jena, Germany) using the NucleoMag® NGS Clean-up and Size Select Kit (Machery-Nagel, Germany) according to manufacturer's instructions. Some samples were quality checked on a random basis by capillary electrophoresis (QIAxcel Advanced, Qiagen,

Germany). Afterwards, sample DNA concentrations were measured with picogreen fluorescence (QuantiFluor® dsDNA System, Promega, USA) on a plate reader (Tecan F200+, Tecan, Switzerland). All samples were normalized to equimolar DNA amounts in a final pool. This allowed us to pool samples in the final library and reliable amplifying the *Cytb* locus from n = 1,172 *Hipposideros caffer* DNA samples. The samples were then sent off to be sequenced by the external next generation sequencing DFG core facility in Bonn, Germany. A paired-end sequencing run was performed on an Illumina MiSeq machine by using the Illumina Reagent Kit V2 chemistry for 300 cycles.

Details on RNA purification and CoV characterization

RNA was purified from approximately 20 mg of faecal material suspended in 500 µl RNAlater stabilizing solution using the MagNA Pure 96 system (Roche, Penzberg, Germany). Elution volumes were set at 100 µl. We used a real-time reverse transcription-PCR (RT-PCR) assay designed to detect bat alpha- and beta-CoVs as described previously[11,12]: For real-time RT-PCR a 25 µl reaction was set up containing 5 µl of RNA, 12.5 µl of the reaction buffer provided with the kit, 1 µL enzyme mix, 0.4 µl of a 50 mM magnesium sulfate solution), 1µg of PCR-grade bovine serum albumin, 400 nM of each of the primers (Supplementary Table 6), as well as 100 nM of each probe. The SSIII RT-PCR kit (Life Technologies, Karlsruhe, Germany) was used together with the following thermal cycling protocol in a LightCycler 480 (Roche, Penzberg, Germany): 20 min at 50°C for reverse transcription, followed by 3 min at 95°C and 45 cycles of 15 s at 95°C, 10 s at 58°C, and 20 s at 72°C. CoV-RNA detection and corresponding Ct values were recorded. In each PCR run, we used *in vitro* transcribed and photometrically quantified RNAs (IVTs, generated from TA-cloned periamplicons using the T7-driven MEGAscript (Life Technologies, Heidelberg, Germany) as positive controls and as calibrators to control for run-to-run consistency[13]. We applied two controls per assay per run: One with a Ct value of ~30 and a second one with a Ct value of ~34 to 38. Only runs with no

or a Ct value shift of the calibrators less than 3 were taken to be valid. We only considered samples with Ct values of 38.0 or less to be CoV positive (equivalent to >15 IVT copies/µL) in order to use only samples with a technically reliable detection within the dynamic range of the real-time RT-PCRs, and to reduce interference from possible contamination in the field. This Ct value was estimated considering the overall RT-PCR sensitivity limits, the Ct value distribution, and the performance of the low concentration positive controls used in each run (Ct value ~34-38). Bats were considered CoV infected if any of the four virus clades were detected in the RT-PCR. The CoV prevalence was estimated as the proportion of bats with a positive RT-PCR result per site and sampling period. All virus PCR data was generated within the same laboratory under carefully controlled and consistent conditions. This and the use of calibrators ensures the comparability of Ct values.

Supplementary Table 6. Primers used for PCR-based detection of bat coronaviruses in Ghana. BetaBI and BetaBII is referred to in text as CoV 2b or 2bBasal, while BetaC is also referred to as CoV 2c.

Primer ID	Sequence (5' - 3')	Polarity
CoV-Hip-BetaBII-rtF	CAGGACGCRCTATTCGCTTA	+
CoV-Hip-BetaBII-rtP	JOE-CGAAGCGTAATGTGTTGCCCACCATAA-BHQ1	+ (Probe)
CoV-Hip-BetaBII-rtR	TGCGCTTATAGCGTATTTCAAATT	-
BetaC-rtF	GCACTGTTGCTGGTGTCTCTATTCT	+
BetaC-rtP	JOE-TGACAAATCGCCAATACCATCAAAAGATGC-BHQ1	+ (Probe)
BetaCrtR	GCCTCTAGTGGCAGCCATACTT	-
CoV-Hip-BetaBI-rt-F	TGCCTAATATGTTGCGTATTTTCG	+
CoV-Hip-BetaBI-rt-P	FAM-TCATTAATYTTGGCTCGTAAGCACTCGACG-BHQ1	+ (Probe)
CoV-Hip-BetaBI-rt-R	ATARTATCGCTCACTCARCGTACAA	-
CoV-Alpha229E-F13948m	TCYAGAGAGGTKGTTGTTACWAAYCT	+
CoV-Alpha229E - P13990m	FAM-TGGCMACTTAATAAGTTTGGIAARGCYGG-BHQ1	+ (Probe)
CoV-Alpha229E - R14138m	CGYTCYTTRCCAGAWATGGCRTA	-

ID - identification; R=G/A, Y=C/T, S=G/C, W=A/T, M=A/C, K=G/T

Phylogenetic analysis of CoVs

RNA-dependent RNA polymerase (RdRp) gene sequences of different bat CoVs as well as zoonotic human CoVs with the respective intermediate host isolates were extracted from genomes deposited in NCBI database using BLAST (for accession numbers see Supplementary Fig. 1). The RdRp sequences were trimmed to an equal length of 367 bp and multiple sequence alignment of the fragments was carried out using the MAFFT plugin in Geneious 11.1.5 (https://www.geneious.com). Bayesian phylogenetic reconstructions for partial RdRp gene sequences were made using MrBayes 3.2.7[14] using gamma-distributed site-specific general time-reversible models. We ran two runs of two chains for ten million MCMC generations with trees sampled every 10,000 steps of which 25% were discarded as burn-in before visualization using FigTree 1.4.4 (http://tree.bio.ed.ac.uk/software/figtree/). All CoV sequences determined in this study were submitted to GenBank under accession numbers OR482956 to OR482966.

2. Supplementary Results

Supplementary Table 7. Distribution of adult (large bat icon) and subadult (small bat icon) captures per species. Icons were created with BioRender.com and coloured by species: *Coleura afra* (slate grey), *Hipposideros (H.) abae* (apricot), *H. caffer B* (light blue), *C* (yellow), *D* (dark blue), *H. jonesi* (blown), *Lissonycteris angolensis* (blue), *Macronycteris gigas* (red), *Nycteris macrotis* (orange), *Rhinolophus landeri* (teal) and *Rousettus aegyptiacus* (olive).

**Species**		***Age***	***n***	***CoV 229E-like (%)***	***CoV 2b (%)***	***CoV 2bBasal (%)***	***CoV 2c (%)***
Coleura afra		Adult	143	0 (0.00)	0 (0.00)	0 (0.00)	0 (0.00)
		Subadult	8	0 (0.00)	0 (0.00)	0 (0.00)	0 (0.00)
Hipposideros abae		Adult	634	27 (4.26)	39 (6.15)	2 (0.32)	0 (0.00)
		Subadult	71	9 (12.68)	20 (28.17)	0 (0.00)	0 (0.00)
Hipposideros caffer B		Adult	116	13 (11.21)	15 (12.93)	0 (0.00)	0 (0.00)
		Subadult	20	5 (25.00)	5 (25.00)	0 (0.00)	0 (0.00)
Hipposideros caffer C		Adult	92	20 (21.74)	14 (15.22)	0 (0.00)	0 (0.00)
		Subadult	31	18 (58.06)	7 (22.58)	0 (0.00)	0 (0.00)
	?	NA	1	1 (100.00)	0 (0.00)	0 (0.00)	0 (0.00)
Hipposideros caffer D		Adult	692	146 (21.10)	320 (46.24)	171 (24.71)	0 (0.00)
		Subadult	214	75 (35.05)	149 (69.63)	19 (8.88)	0 (0.00)
	?	NA	1	0 (0.00)	0 (0.00)	0 (0.00)	0 (0.00)
Hipposideros jonesi		Adult	32	0 (0.00)	1 (3.13)	0 (0.00)	0 (0.00)
		Subadult	6	0 (0.00)	1 (16.67)	0 (0.00)	0 (0.00)
Lissonycteris angolensis		Adult	13	0 (0.00)	1 (7.69)	0 (0.00)	0 (0.00)
		Subadult	6	0 (0.00)	0 (0.00)	0 (0.00)	0 (0.00)
Macronycteris gigas		Adult	29	0 (0.00)	1 (3.45)	1 (3.45)	1 (3.45)
		Subadult	13	0 (0.00)	0 (0.00)	0 (0.00)	0 (0.00)
	?	NA	2	0 (0.00)	0 (0.00)	0 (0.00)	0 (0.00)
Nycteris macrotis		Adult	202	0 (0.00)	1 (0.50)	0 (0.00)	26 (12.87)
		Subadult	19	0 (0.00)	0 (0.00)	0 (0.00)	4 (21.05)
Rhinolophus landeri		Adult	11	0 (0.00)	0 (0.00)	0 (0.00)	0 (0.00)
		Subadult	4	0 (0.00)	1 (25.00)	0 (0.00)	0 (0.00)
Rousettus aegyptiacus		Adult	2	0 (0.00)	0 (0.00)	0 (0.00)	0 (0.00)
		Subadult	0	0 (0.00)	0 (0.00)	0 (0.00)	0 (0.00)
Total			2362	314 (13.29)	575 (24.34)	193 (8.17)	31 (1.31)

Supplementary Table 8. Summary of the Generalized Linear Model (GLM) analysis for predicting coronavirus infection likelihood. Presented are results from a GLM examining the relationship between individual bat infection and species identity and age using the complete dataset (n = 2,358; four bats had no age information). Icons were created with BioRender.com and coloured by species: *Coleura afra* (slate grey), *Hipposideros (H.) abae* (apricot), *H. caffer B* (light blue), *C* (yellow), *D* (dark blue), *H. jonesi* (blown), *Lissonycteris angolensis* (blue), *Macronycteris gigas* (red), *Nycteris macrotis* (orange), *Rhinolophus landeri* (teal) and *Rousettus aegyptiacus* (olive); or age: subadults (small pink icon).

	Predictors	***Estimates***	***CI***	***p-value***
**alpha-CoV 229E-like**	*CoV 229E-like ~ Species + age*			
	(Intercept)	-0.01	-0.06 – 0.04	0.800
	Hipposideros abae	0.05	-0.01 – 0.10	0.114
	Hipposideros caffer B	0.12	0.05 – 0.19	**0.001**
	Hipposideros caffer C	0.28	0.21 – 0.36	**<0.001**
	Hipposideros caffer D	0.22	0.17 – 0.28	**<0.001**
	Hipposideros jonesi	-0.01	-0.13 – 0.10	0.822
	Lissonycteris angolensis	-0.03	-0.18 – 0.12	0.674
	Macronycteris gigas	-0.03	-0.14 – 0.08	0.568
	Nycteris macrotis	-0.00	-0.07 – 0.06	0.903
	Rhinolophus landeri	-0.03	-0.20 – 0.14	0.759
	Rousettus aegyptiacus	0.01	-0.44 – 0.45	0.977
	Subadult	0.12	0.09 – 0.16	**<0.001**
	Observations	2358		
	R^2	0.122		
**beta-CoV 2b**	*CoV 2b ~ Species + age*			
	(Intercept)	-0.01	-0.07 – 0.05	0.746
	Hipposideros abae	0.08	0.01 – 0.14	**0.021**
	Hipposideros caffer B	0.13	0.05 – 0.21	**0.003**
	Hipposideros caffer C	0.13	0.05 – 0.22	**0.002**
	Hipposideros caffer D	0.48	0.42 – 0.55	**<0.001**
	Hipposideros jonesi	0.03	-0.10 – 0.16	0.610
	Lissonycteris angolensis	0.01	-0.17 – 0.18	0.954
	Macronycteris gigas	-0.02	-0.15 – 0.10	0.722
	Nycteris macrotis	-0.00	-0.08 – 0.07	0.970
	Rhinolophus landeri	0.03	-0.17 – 0.22	0.776
	Rousettus aegyptiacus	0.01	-0.50 – 0.52	0.970
	Subadult	0.18	0.14 – 0.22	**<0.001**
	Observations	2358		
	R^2	0.286		

Supplementary Table 9. Summary of the Generalized Linear Model (GLM) analysis for predicting coronavirus infection likelihood. Presented are results from a GLM examining the relationship between individual bat infection and species identity focusing on the *Hipposideros* subset (n = 1,908). Icons were created with BioRender.com and coloured by species: *Hipposideros (H.) caffer B* (light blue), *C* (yellow), *D* (dark blue), *H. jonesi* (blown).

	Predictors	***Estimates***	***CI***	***p-value***
**alpha-CoV 229E-like**	*CoV 229E-like ~ Species within Hipposideros genus*			
	(Intercept)	0.05	0.02 – 0.08	**<0.001**
	Hipposideros caffer B	0.08	0.02 – 0.15	**0.015**
	Hipposideros caffer C	0.26	0.19 – 0.33	**<0.001**
	Hipposideros caffer D	0.19	0.16 – 0.23	**<0.001**
	Hipposideros jonesi	-0.05	-0.17 – 0.07	0.391
	Observations	1908		
	R^2	0.071		
**beta-CoV 2b**	*CoV 2b ~ Species within Hipposideros genus*			
	(Intercept)	0.08	0.05 – 0.11	**<0.001**
	Hipposideros caffer B	0.06	-0.01 – 0.14	0.097
	Hipposideros caffer C	0.09	0.01 – 0.17	**0.029**
	Hipposideros caffer D	0.43	0.39 – 0.47	**<0.001**
	Hipposideros jonesi	-0.03	-0.16 – 0.10	0.648
	Observations	1908		
	R^2	0.209		

Supplementary Figure 1

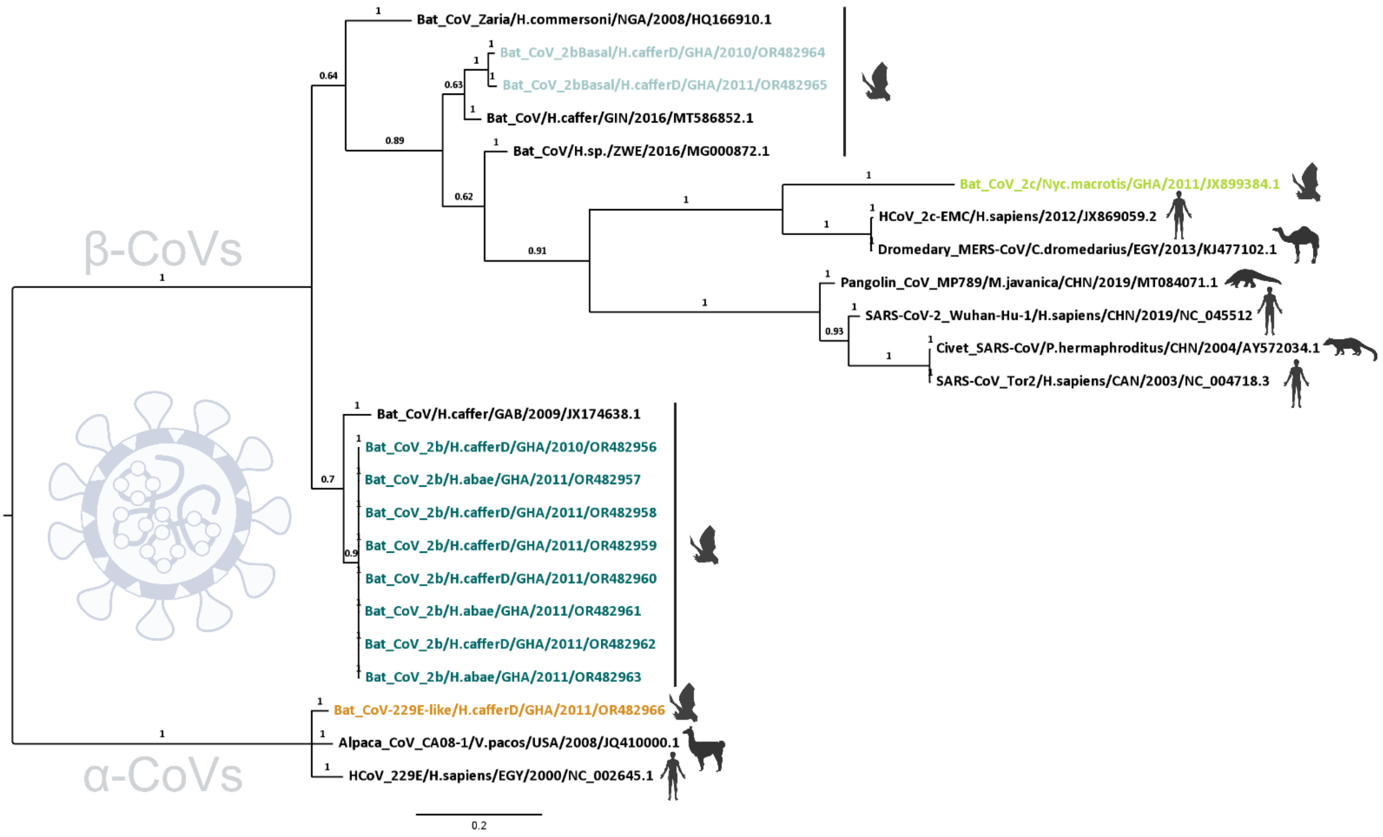

Supplementary Figure 1. Phylogenetic reconstruction of all four bat coronaviruses (CoVs) investigated in this study depicted in colour. The figure shows a Bayesian phylogeny of RNA-dependent RNA polymerases (RdRp) fragments (including accession numbers downloaded from GenBank), the bar represents the estimated number of nucleotide substitutions per site. Posterior support values are indicated on the branches. Icons were created with BioRender.com.

Supplementary Figure 2

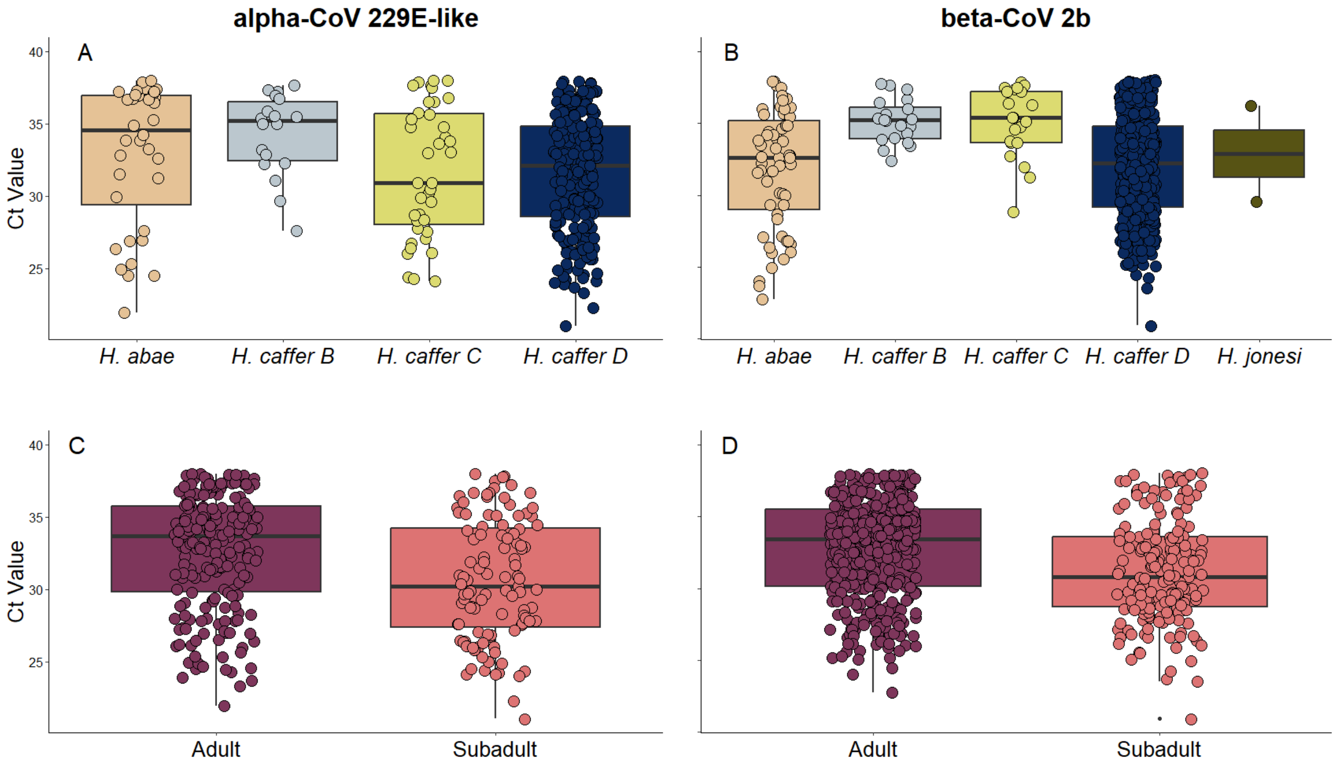

Supplementary Figure 2. Differences in cycle threshold (Ct) values between *Hipposideros* species (A: $p = 0.023$ for alpha-CoV 229E-like and B: $p < 0.001$ for beta-CoV 2b) as well as adult and subadult bats (C and D: $p < 0.001$ for both CoVs) infected with the alpha-CoV 229E-like (A and C) or beta-CoV 2b (B and D). Infection was defined as RT-qPCR-positive if the Ct value was below the threshold of 38 cycles.

Supplementary Figure 3

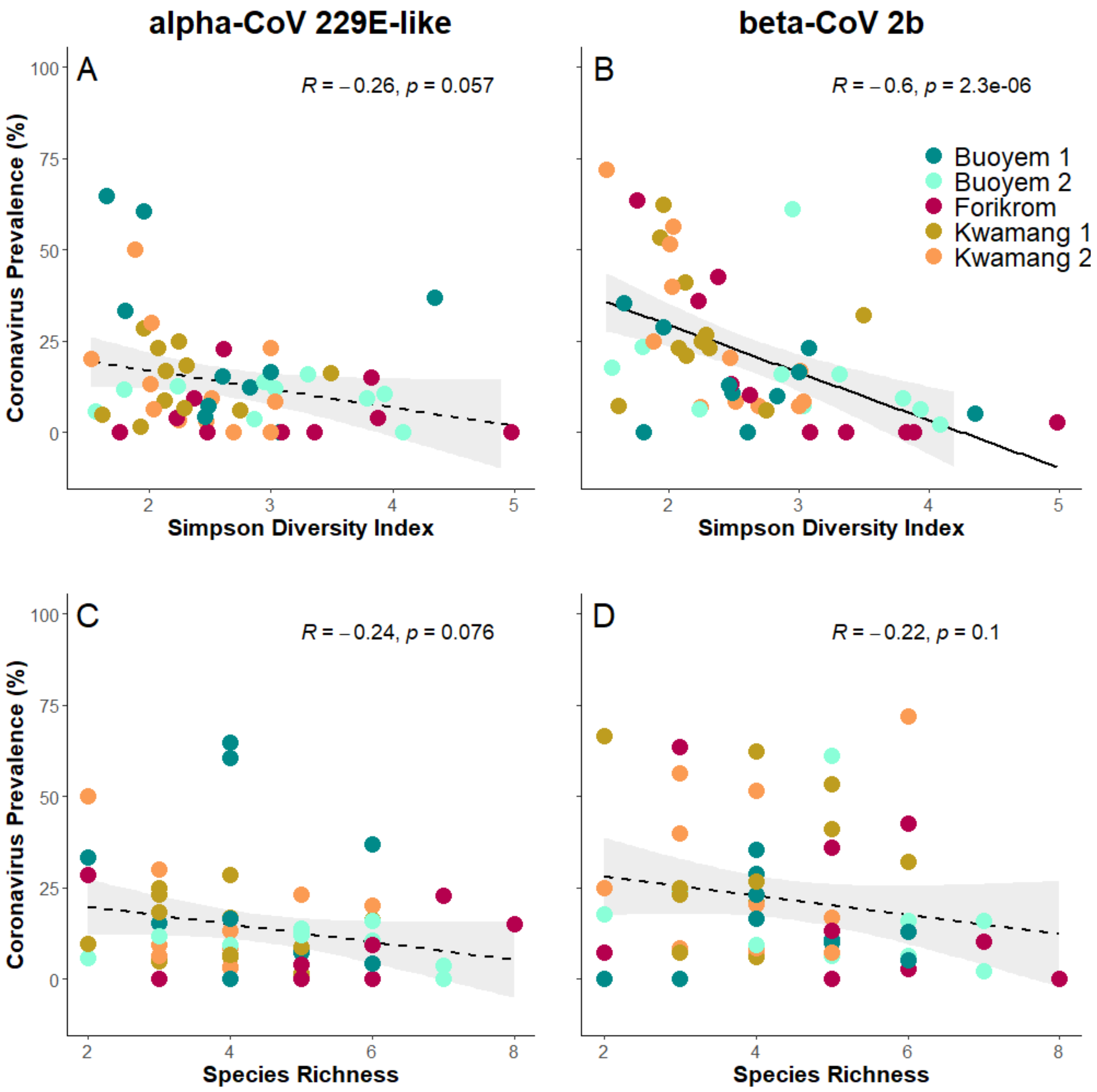

Supplementary Figure 3. Spearman-correlation between coronavirus prevalence (alpha-CoV 229E-like, beta-CoV 2b) and the Simpson Diversity Index (A, B) or species richness (C, D). Black line and grey band show best fit line and 95 percent confidence interval, respectively.

3. Supplementary Reference List

1 Nkrumah, E. E. *et al.* Diversity and Conservation of Cave-Roosting Bats in Central Ghana. *Trop Conserv Sci* **14**, doi:Artn 19400829211034671
10.1177/19400829211034671 (2021).

2 Vallo, P., Guillen-Servent, A., Benda, P., Pires, D. B. & Koubek, P. Variation of mitochondrial DNA in the Hipposideros caffer complex (Chiroptera: Hipposideridae) and its taxonomic implications. *Acta Chiropterol* **10**, 193-206, doi:10.3161/150811008x414782 (2008).

3 Baldwin, H. J. *et al.* Concordant patterns of genetic, acoustic, and morphological divergence in the West African Old World leaf-nosed bats of the Hipposideros caffer complex. *J Zool Syst Evol Res* **59**, 1390-1407, doi:10.1111/jzs.12506 (2021).

4 Tanalgo, K. C., Tabora, J. A. G. & Hughes, A. C. Bat cave vulnerability index (BCVI): A holistic rapid assessment tool to identify priorities for effective cave conservation in the tropics. *Ecol Indic* **89**, 852-860, doi:10.1016/j.ecolind.2017.11.064 (2018).

5 Tanalgo, K. C., Oliveira, H. F. M. & Hughes, A. C. Mapping global conservation priorities and habitat vulnerabilities for cave-dwelling bats in a changing world. *Sci Total Environ* **843**, 156909, doi:10.1016/j.scitotenv.2022.156909 (2022).

6 Anti, P. *et al.* Human-Bat Interactions in Rural West Africa. *Emerg Infect Dis* **21**, 1418-1421, doi:10.3201/eid2108.142015 (2015).

7 Theobald, D. M. *et al.* Earth transformed: detailed mapping of global human modification from 1990 to 2017. *Earth Syst Sci Data* **12**, 1953-1972, doi:10.5194/essd-12-1953-2020 (2020).

8 Theobald, D. M., Kennedy, Christina, Chen, Bin, Oakleaf, James, Baruch-Mordo, Sharon, & Kiesecker, Joe. (Zenodo, 2023).

9 Bradley, R. D. & Baker, R. J. A test of the genetic species concept: Cytochrome-b sequences and mammals. *J Mammal* **82**, 960-973, doi:Doi 10.1644/1545-1542(2001)082<0960:Atotgs>2.0.Co;2 (2001).

10 Nicholls, J. A., Double, M. C., Rowell, D. M. & Magrath, R. D. The evolution of cooperative and pair breeding in thornbills Acanthiza (Pardalotidae). *J Avian Biol* **31**, 165-176, doi:DOI 10.1034/j.1600-048X.2000.310208.x (2000).

11 Corman, V. M. *et al.* Evidence for an Ancestral Association of Human Coronavirus 229E with Bats. *J Virol* **89**, 11858-11870, doi:10.1128/JVI.01755-15 (2015).

12 Pfefferle, S. *et al.* Distant Relatives of Severe Acute Respiratory Syndrome Coronavirus and Close Relatives of Human Coronavirus 229E in Bats, Ghana. *Emerging Infectious Diseases* **15**, 1377-1384, doi:10.3201/eid1509.090224 (2009).

13 Drexler, J. F. *et al.* A novel diagnostic target in the hepatitis C virus genome. *PLoS Med* **6**, e31, doi:10.1371/journal.pmed.1000031 (2009).

14 Ronquist, F. & Huelsenbeck, J. P. MrBayes 3: Bayesian phylogenetic inference under mixed models. *Bioinformatics* **19**, 1572-1574, doi:10.1093/bioinformatics/btg180 (2003).
