# Supplementary material for: Bat species assemblage predicts coronavirus prevalence
Source: Nat Commun. 2024 Apr 4;15:2887. doi: 10.1038/s41467-024-46979-1 (PMC10994947; doi:10.1038/s41467-024-46979-1)
Supplement: Supplementary file 3 — Description of Additional Supplementary Files [file 41467_2024_46979_MOESM3_ESM.pdf]

Description of Additional Supplementary Files

Title: Supplementary Data 1

Description: Captured individuals and subadults per species across sampling site and period.

Title: Supplementary Data 2

Description: Diversity indices per sampling site and period.

Title: Supplementary Data 3

Description: Dredge summary for all equally competitive models (ΔAICC ≤ 2.00) predicting infection probability with either alpha-CoV 229E-like or beta-CoV 2b. Listed is model structure, Akaike's information criterion (AICC) values, the difference in AICc to other models falling within ΔAICC ≤ 2.00, and Akaike weights representing the probability that a given model is the best approximating model. Framed are dredge results for models that included different bat species relative abundances. For the sake of comparability, the results in the main text, summary tables and visuals report the results from the full model containing all explanatory variables (if the full model was competitive, which was always the case). Model averaging was performed for all models with ΔAICC ≤ 2.00 (see Supplementary Data 7-9), but never changed the interpretation of the results.

Title: Supplementary Data 4

Description: Summary table of the results from the full generalised linear mixed effect models comparing the effect of Shannon Diversity Index and the abundance of the host species *Hipposideros* (*H.*) *abae*, *caffer B*, *C* and *D*, *C. afra* and *N. macrotis* as well as subadults on coronavirus (CoV) infection probability that are not shown in Table 2 in the main text. Significant results are in bold and are presented as original and FDR-corrected p-values.

Title: Supplementary Data 5

Description: Summary table of the results from the from the full generalised linear mixed effect models comparing the effect of Simpson Diversity Index and the abundance of the host species *Hipposideros (H.) abae, caffer B, C* and *D, C. afra* and *N. macrotis* as well as subadults on coronavirus (CoV) infection probability that are not shown in Table 2 in the main text. Significant results are in bold and are presented as original and FDR-corrected p-values.

Title: Supplementary Data 6

Description: Summary table of the results from the full generalised linear mixed effect models comparing the effect of Species Richness and the abundance of the host species *Hipposideros (H.) abae, caffer B, C* and *D, C. afra* and *N. macrotis* as well as subadults on coronavirus (CoV) infection probability that are not shown in Table 2 in the main text. Significant results are in bold and are presented as original and FDR-corrected p-values.

Title: Supplementary Data 7

Description: Summary table of the results from the model averages comparing the effect of Shannon Diversity Index and the abundance of the host species *Hipposideros (H.) abae, caffer B, C* and *D, C. afra* and *N. macrotis* as well as subadults on coronavirus (CoV) infection probability. Model averaging was completed for generalised linear mixed effect models falling within $\Delta AIC_C \leq 2.0$ presented in Supplementary Data 3. Significant results are in bold and are presented as original and FDR-corrected p-values.

Title: Supplementary Data 8

Description: Summary table of the results from the model averages comparing the effect of Simpson Diversity Index and the abundance of the host species *Hipposideros (H.) abae, caffer B, C* and *D, C. afra* and *N. macrotis* as well as subadults on coronavirus (CoV) infection probability. Model averaging was completed for generalised linear mixed effect models

falling within $\Delta AIC_C \leq 2.0$ presented in Supplementary Data 3. Significant results are in bold and are presented as original and FDR-corrected p-values.

Title: Supplementary Data 9

Description: Summary table of the results from the model averages comparing the effect of Species Richness and the abundance of the host species *Hipposideros (H.) abae, caffer B, C* and *D, C. afra* and *N. macrotis* as well as subadults on coronavirus (CoV) infection probability that are not shown in Table 2 in the main text. Model averaging was completed for generalised mixed effect models falling within $\Delta AIC_C \leq 2.0$ presented in Supplementary Data 3. Significant results are in bold and are presented as original and FDR-corrected p-values.
