# Supplementary material for: Bat species assemblage predicts coronavirus prevalence
Source: Nat Commun. 2024 Apr 4;15:2887. doi: 10.1038/s41467-024-46979-1 (PMC10994947; doi:10.1038/s41467-024-46979-1)
Supplement: Supplementary file 13 — Reporting Summary [file 41467_2024_46979_MOESM13_ESM.pdf]

nature portfolio

Corresponding author(s): Magdalena Meyer and Simone Sommer

Last updated by author(s): Feb 21, 2024

Reporting Summary

Nature Portfolio wishes to improve the reproducibility of the work that we publish. This form provides structure for consistency and transparency in reporting. For further information on Nature Portfolio policies, see our Editorial Policies and the Editorial Policy Checklist.

Statistics

For all statistical analyses, confirm that the following items are present in the figure legend, table legend, main text, or Methods section.

n/a	Confirmed	
☐	☒	The exact sample size (*n*) for each experimental group/condition, given as a discrete number and unit of measurement
☐	☒	A statement on whether measurements were taken from distinct samples or whether the same sample was measured repeatedly
☐	☒	The statistical test(s) used AND whether they are one- or two-sided<br>*Only common tests should be described solely by name; describe more complex techniques in the Methods section.*
☐	☒	A description of all covariates tested
☐	☒	A description of any assumptions or corrections, such as tests of normality and adjustment for multiple comparisons
☐	☒	A full description of the statistical parameters including central tendency (e.g. means) or other basic estimates (e.g. regression coefficient) AND variation (e.g. standard deviation) or associated estimates of uncertainty (e.g. confidence intervals)
☐	☒	For null hypothesis testing, the test statistic (e.g. *F*, *t*, *r*) with confidence intervals, effect sizes, degrees of freedom and *P* value noted<br>*Give P values as exact values whenever suitable.*
☐	☒	For Bayesian analysis, information on the choice of priors and Markov chain Monte Carlo settings
☐	☒	For hierarchical and complex designs, identification of the appropriate level for tests and full reporting of outcomes
☐	☒	Estimates of effect sizes (e.g. Cohen's *d*, Pearson's *r*), indicating how they were calculated

Our web collection on statistics for biologists contains articles on many of the points above.

Software and code

Policy information about availability of computer code

Data collection	No software was used for data collection.
Data analysis	Bioinformatic and statistical analyses were performed in the software R (v4.1.1; R Core Team 2022). The R code including all packages used for the analyses of the current study is available on GitHub: https://github.com/MagdalenaMeyer/Bat-species-assemblage-predicts-CoV-prevalence as well as figshare: Meyer, Magdalena; Schmid, Dominik Werner; Sommer, Simone (2023): Bat species assemblage predicts coronavirus prevalence. figshare. Dataset. https://doi.org/10.6084/m9.figshare.21982592. Assignment of bat species, trimming of CoV RdRp sequences and their alignment was completed in Geneious 11.1.5 (https://www.geneious.com) using the MAFFT alignment tool. Bayesian phylogenetic reconstructions for partial RdRp gene sequences were made using MrBayes 3.2.7. Visualisation of the phylogenetic analysis of CoVs was performed using FigTree 1.4.4.

For manuscripts utilizing custom algorithms or software that are central to the research but not yet described in published literature, software must be made available to editors and reviewers. We strongly encourage code deposition in a community repository (e.g. GitHub). See the Nature Portfolio guidelines for submitting code & software for further information.

Data

Policy information about availability of data

All manuscripts must include a data availability statement. This statement should provide the following information, where applicable:

- Accession codes, unique identifiers, or web links for publicly available datasets
- A description of any restrictions on data availability
- For clinical datasets or third party data, please ensure that the statement adheres to our policy

The dataset generated and/or analyzed during the current study is available on GitHub:
https://github.com/MagdalenaMeyer/Bat-species-assemblage-predicts-CoV-prevalence
...and figshare:
Meyer, Magdalena; Schmid, Dominik Werner; Sommer, Simone (2023): Bat species assemblage predicts coronavirus prevalence. figshare. Dataset. https://doi.org/10.6084/m9.figshare.21982592

Research involving human participants, their data, or biological material

Policy information about studies with human participants or human data. See also policy information about sex, gender (identity/presentation), and sexual orientation and race, ethnicity and racism.

Reporting on sex and gender	N/A
Reporting on race, ethnicity, or other socially relevant groupings	N/A
Population characteristics	N/A
Recruitment	N/A
Ethics oversight	N/A

Note that full information on the approval of the study protocol must also be provided in the manuscript.

Field-specific reporting

Please select the one below that is the best fit for your research. If you are not sure, read the appropriate sections before making your selection.

☐ Life sciences ☐ Behavioural & social sciences ☒ Ecological, evolutionary & environmental sciences

For a reference copy of the document with all sections, see nature.com/documents/nr-reporting-summary-flat.pdf

Ecological, evolutionary & environmental sciences study design

All studies must disclose on these points even when the disclosure is negative.

Study description	For our longitudinal study to investigate the community assemblage of Ghanaian bats in relation to infections with coronaviruses, more than 14,000 bats were captured at 17 sites in Ghana, West Africa, between August 2010 and August 2012. While some sites were sampled only sporadically, five different caves in central Ghana formed the core of the sampling effort. These were sampled every two months and are the major focus of analysis in this manuscript. Bat species were identified based on morphological or, in the case of cryptic species complexes, genetic characteristics (mitochondrial cytb markers). The overall recapture rate of less than 0.25% allowed each sampling site and period to be treated as an independent sample, which meant that no correction was required to account for four missing sampling dates. Using the collected fecal samples, bats were tested for four different coronavirus (CoV) strains. On this basis, infection data for single individuals as well as prevalences for roosting communities could be determined. The objective of the study was to investigate the effects of community composition and demographic structure on CoV infection probabilities and prevalences. We tested the correlation between CoV prevalence and species diversity using Spearman's correlation and applied generalized mixed-effects linear models to estimate CoV infection probability as a binomial response variable (positive/negative coded as 1/0) with one of the diversity indices (species richness, Shannon or Simpson index), relative abundance of common host species and relative abundance of subadult bats as explanatory variables, and sampling period (n=12) nested within the sampling site (n=5) as a random effect.
Research sample	The research samples used in this study were faecal samples and 2mm wing punches of bat species communities sharing roosts in various caves in Ghana. The following bat species were captured in the caves: Coleura afra, Hipposideros abae, Hipposideros caffer B, Hipposideros caffer C, Hipposideros caffer D, Hipposideros jonesi, Lissonycteris angolensis, Macronycteris gigas, Nycteris macrotis, Rhinolophus landeri and Rousettus aegyptiacus. The age range of the animals included juveniles capable of flight, i.e., subadults, and adult bats.

Sampling strategy

To standardize the sampling effort for each site and sampling period, bats were trapped at each cave roost for two nights with mist nets stretched along cave entrances one hour after dark until dawn. Thus, the number of bats captured at each time point under the same conditions is representative of the size of the roosting community, which is subject to natural variation.

Data collection

HJB and MT organized the field work. EEN, EKB, SKO, PV, MT and HJB collected field data and archived biological samples. KW, DWS and MM completed laboratory work for lineage assignment based on previous work from HJB, AS and PV. HJB, VMC and CD generated the viral infection data. MM, DWS and KW analysed data.

Timing and spatial scale

Fieldwork was conducted over two years from August 2010 to August 2012 in various locations in Ghana, West Africa. Individual sites differed in their sampling effort and ranged from opportunistic to semiannual to core sites sampled bimonthly. There are five missing time points for the core sampling sites, which are Nov-Dec 2010 & Jan-Feb 2011 for Buoyem 1 and 2 as well as Nov-Dec 2010 for Forikrom.

Data exclusions

No data were excluded from the analyses.

Reproducibility

The extensive fieldwork that resulted in an enormous data set during the course of this study allowed for verification of the results at different locations over different time points.

Randomization

For the study of roosting communities and prevalences, bats were assigned based on their capture location at each time point. For the study of infection probabilities, animals were considered individually based on their infection status.

Blinding

All laboratory work was performed blind.

Did the study involve field work? ☒ Yes ☐ No

Field work, collection and transport

Field conditions

Ghana is a country with a tropical climate, an alternation between rainy and dry seasons, and different vegetation zones from north to south. During the two years of fieldwork, temperature and precipitation variations were observed at the different sample sites, however it was not part of the study to record exact climate parameters.

Location

17 sample sites during the two-year field work in Ghana, West Africa.
Mines or buildings were abandoned and open represents forested or farmed land as sampling site.

Site / Site type / Latitude / Longitude / Sampling regime

Akpafu Todzi / mine / 7.2619722 / 0.4915278 / semiannual
Bobiri / open / 6.6871 / -1.34415 / opportunistic
Botanical Gardens / open / 6.6851111 / -1.5618889 / opportunistic
Lake Bosomtwe / open / 6.5395278 / -1.4115278 / opportunistic
Buoyem 1 / cave / 7.7235833 / -1.9879167 / bimonthly
Buoyem 2 / cave / 7.7238056 / -1.9926389 / bimonthly
Elmina / building / 5.0827778 / -1.3483056 / semiannual
Forikrom / cave / 7.58975 / -1.8750833 / bimonthly
Kumasi Centre for Collaborative Research in Tropical Medicine / open / 6.6698226 / -1.5771767 / opportunistic
Kwamang 1 / cave / 7.0035685 / -1.3003098 / bimonthly
Kwamang 2 / cave / 6.9832778 / -1.2731944 / bimonthly
Kwamang 3 / open / 7.0065315 / -1.3012354 / opportunistic
Likpe Todome 1 / cave / 7.1639444 / 0.6079167 / semiannual
Likpe Todome 2 / cave / 7.1638611 / 0.6081389 / semiannual
Shai Hills / cave / 5.9290000 / 0.0750000 / opportunistic
University Cape Coast / open / 5.1202112 / -1.2935219 / opportunistic
Bui / open / 8.3893458 / -2.3813621 / opportunistic

Access & import/export

Official permission for capturing and sampling was obtained from the Wildlife Division of the Forestry Commission of the Ministry of Lands, Forestry and Mines, under research permit A04957 and ethics permit CHRPE49/09/CITES. All animals were handled according to the European Union Council Directive 86/609/EEC for the protection of animals and in accordance with Ghanaian law. In addition, permits were obtained from the local chiefs of the villages near the caves.

Disturbance

To minimize disturbance to the bats, a break between the first and second sampling night was enforced. To ensure the welfare of the animals during capture, the peak activity period of the bats during netting was avoided so that the animals could be released as soon as possible, but within a maximum of two hours.

Reporting for specific materials, systems and methods

We require information from authors about some types of materials, experimental systems and methods used in many studies. Here, indicate whether each material, system or method listed is relevant to your study. If you are not sure if a list item applies to your research, read the appropriate section before selecting a response.

Materials & experimental systems

n/a	Involved in the study
☒	☐ Antibodies
☒	☐ Eukaryotic cell lines
☒	☐ Palaeontology and archaeology
☐	☒ Animals and other organisms
☒	☐ Clinical data
☒	☐ Dual use research of concern
☒	☐ Plants

Methods

n/a	Involved in the study
☒	☐ ChIP-seq
☒	☐ Flow cytometry
☒	☐ MRI-based neuroimaging

Animals and other research organisms

Policy information about studies involving animals; ARRIVE guidelines recommended for reporting animal research, and Sex and Gender in Research

Laboratory animals	The study did not involve laboratory animals.
Wild animals	Bats were trapped in each cave for two nights with mist nets stretched along cave entrances one hour after dark until dawn. Captured bats were temporarily held in cloth bags and processed within 2 hours. They were then released promptly at the capture site immediately after data recording and sample collection, ensuring release within a maximum of 2 hours. The following cave-dwelling bat species were captured: Coleura afra, Hipposideros abae, Hipposideros caffer B, Hipposideros caffer C, Hipposideros caffer D, Hipposideros jonesi, Lissonycteris angolensis, Macronycteris gigas, Nycteris macrotis, Rhinolophus landeri, and Rousettus aegyptiacus. The age range of the animals included flight-capable juveniles, subadults, and adult bats.
Reporting on sex	The sex of captured bats was determined by sighting and measuring external sexual characteristics (e.g. testis length and width). Of a total of 14,468 captured animals, 6173 were identified as female and 8251 as male. The sex of 44 animals could not be further determined because they escaped before data were collected.<br>A species community-based approach was used in this study, controlling for the influence of characteristics of individual animals. The results of this study are not influenced by the sex of the animals.
Field-collected samples	Minimally invasive wing punches (2 mm) were collected and stored at -20°C, while fecal samples were collected and preserved in RNAlater at -80°C until further processing in the laboratory. No housing of animals was necessary, as they were wild animals captured briefly for non-invasive sample collection purposes. The obtained samples were then promptly transferred to -80°C for long-term storage (Qiagen, Germany). Throughout the transfer process, the frozen samples remained at -80°C, maintaining an uninterrupted cold chain and preventing exposure to light until RNA or DNA extraction, as detailed in the methods section.
Ethics oversight	Official permission for capturing and sampling was obtained from the Wildlife Division of the Forestry Commission of the Ministry of Lands, Forestry and Mines, under ethics permit CHRPE49/09/CITES. All animals were handled according to the European Union Council Directive 86/609/EEC for the protection of animals and in accordance with Ghanaian law.

Note that full information on the approval of the study protocol must also be provided in the manuscript.

Plants

Seed stocks	N/A
Novel plant genotypes	N/A
Authentication	N/A
